# Small Extracellular Vesicle‐Associated Polymeric Immunoglobulin Receptor in Primary Liver Cancer: From Immunological Mediator to Oncogenic Driver and Biomarker

**DOI:** 10.1111/cpr.70243

**Published:** 2026-06-12

**Authors:** Jeremy Ting Ruei Hsu, Samuel Wan Ki Wong, Judy Yam Wai Ping

**Affiliations:** ^1^ Department of Pathology, Li Ka Shing Faculty of Medicine The University of Hong Kong Hong Kong China; ^2^ State Key Laboratory of Liver Research The University of Hong Kong Hong Kong China; ^3^ Materials Innovation Institute for Life Sciences and Energy (MILES) Shenzhen China; ^4^ Department of Hepatopancreatobiliary Surgery, the Second Affiliated Hospital of Harbin Medical University Harbin Heilongjiang China

To the Editor,

Primary liver cancer is a broad term that refers to all malignant neoplasms that arise within the liver, with hepatocellular carcinoma (HCC) and intrahepatic cholangiocarcinoma (CCA) being the two most common [1] Among the two most common types of primary liver cancers, HCC remains the predominant subtype, accounting for more than 80% of all cases [2]. In Hong Kong, HCC has an incidence rate of approximately 16.5 per 100,000 males, ranking as the fourth most common cancer affecting men in the region [3]. Despite its high incidence, the true clinical burden of HCC is largely attributable to its high mortality. HCC is the third leading cause of cancer‐related death worldwide, accounting for around 830,000 deaths annually, with a rising mortality rate. This dismal prognosis is primarily due to HCC's aggressive nature and the frequent late diagnosis, which severely limits effective treatment options. Intrahepatic CCA similarly carries a poor prognosis, largely due to its aggressive biology and frequent diagnosis at advanced stages when curative options are limited. The lack of reliable early diagnostic markers resulted in poor clinical outcomes and underscores the urgent need for novel biomarkers [4].

Extracellular vesicles (EVs) are a broad group of heterogeneous membrane‐bound particles released by cells. Following current guidelines, small EVs (sEVs) are typically sized < 200 nm [5]. These sEVs facilitate intercellular communications by transporting proteins and nucleic acids, thereby shaping the tumour microenvironment and facilitating proliferation, invasion, metastasis, and therapeutic resistance [6]. In HCC, recent evidence has suggested that sEVs play pivotal roles in tumour progression [7]. More interestingly, sEVs have also been shown to be detectable in body fluids even at early stages of disease, positioning them as promising biomarkers and therapeutic targets for multiple cancers [8]. Understanding how sEV cargo influences cancer cell behaviour and metastasis is crucial for advancing early diagnosis and treatment. sEV‐associated polymeric immunoglobulin receptor (sEV‐pIgR) has been demonstrated to drive cancer stemness, tumorigenesis, and metastasis in HCC [9]. Considering sEV‐pIgR as a potential biomarker, this review discusses and invites perspectives regarding its clinical implications in diagnosis, addresses current limitations, and explores future research directions aimed at improving clinical management.

## 1 Established sEV‐pIgR as Functional Oncogenic Component in HCC

A study by Tey et al. demonstrated that circulating sEVs derived from late‐stage HCC patients were more potent than those from early‐stage disease in promoting migration, invasion, sphere formation, in vivo tumorigenesis and metastasis [9]. Proteomic profiling revealed that the circulating sEVs from HCC patients carry significantly higher levels of pIgR compared to those from healthy or chronic liver disease patients [9]. Neutralising antibody against pIgR markedly reduced the pro‐tumour effects of HCC sEVs on cell migration, invasion, and sphere formation, validating pIgR as a key oncogenic component of sEV [9].

**FIGURE 1:**
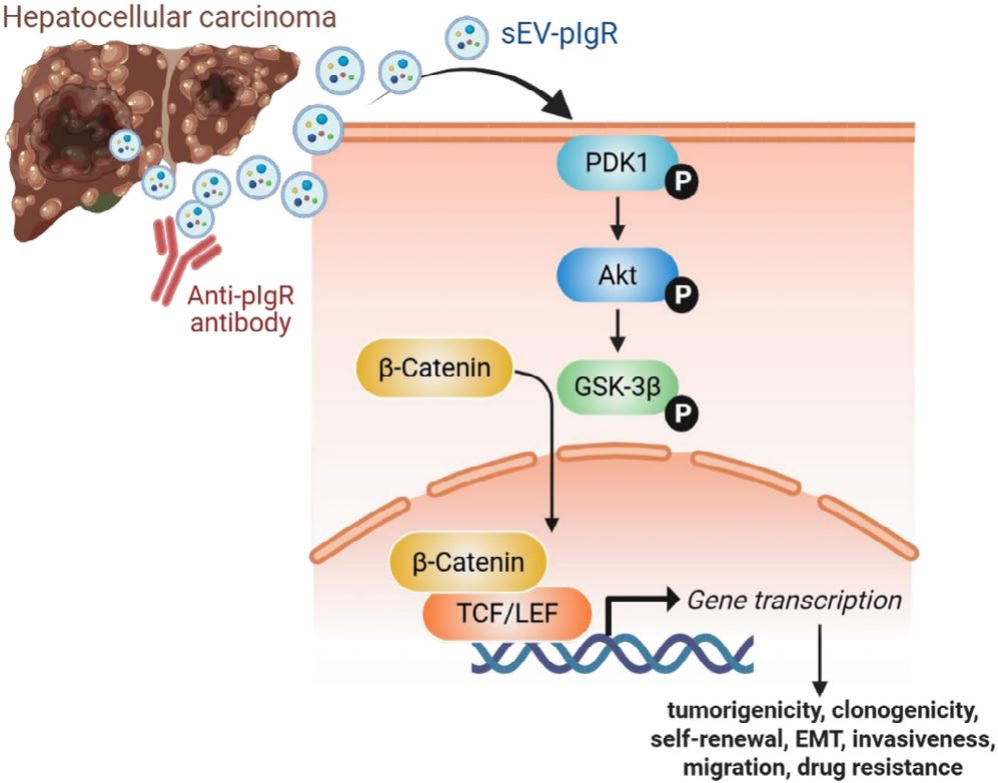
Functions and mechanistic basis of sEV‐pIgR in HCC.

pIgR is traditionally recognised as a transcytosis receptor for dimeric IgA and pentameric IgM, primarily involved in mucosal immunity. Mechanistically, the study proposed that sEV‐pIgR activate the PDK1/Akt/GSK3β/β‐catenin signalling pathway in HCC cells, leading to β‐catenin stabilisation and nuclear translocation (Figure 1), a pathway known to promote cancer stem cell maintenance and metastasis in HCC [9]. Other studies have linked the pathway to the activation of epithelial‐mesenchymal transition (EMT) in HCC [10]– a process known to increase the malignancy of pre‐formed tumours. Apart from established HCC maintenance and increasing its metastatic potential, studies have also proposed the oncogenic potential of pIgR through stimulation of the ribosome pathway, which has been associated with inducing a decrease in p53 stability [11]. It is clear that recent evidence has demonstrated that pIgR's function transcends the orthodox perspective of being a simple immunological mediator, but instead is a complex mediator [12] in HCC oncogenesis, maintenance and metastasis.

## 2 Putative Upstream Drivers of Aberrant Cellular pIgR in Hepatocytes and Their Relevance With Primary Liver Cancer

In HCC, it is plausible that aberrant cellular pIgR expression may reflect the coupling of inflammatory reprogramming of malignant hepatocytes with cytokine‐driven transcriptional induction. Current literature suggests that in healthy human liver, cellular pIgR is primarily expressed in cholangiocytes, yet inflammatory liver injury is associated with increased cellular pIgR accumulation [13]. This indicates that upstream inflammatory cues may be associated with the reactivation of previously silenced pIgR expression in hepatocytes. Studies on biliary atresia have reported that pro‐inflammatory cytokines: IL‐1ß, TNF‐a, and IFN‐y upregulate pIgR expression in cholangiocytes [14].

Although the induction of pIgR under inflammatory conditions raises legitimate concerns about its specificity as a biomarker for primary liver cancer, an important distinction must be made. The studies by Tey et al. and this review focus specifically on sEV‐associated pIgR, rather than cellular pIgR. Notably, sEV‐pIgR and cellular pIgR exhibit a key structural difference: cellular pIgR is glycosylated, whereas sEV‐pIgR is non‐glycosylated [9]. This distinction suggests that the secretory pattern of sEV‐pIgR is not a downstream consequence of cellular pIgR upregulation, but likely requires additional oncogenic signalling or alterations in vesicular trafficking beyond simple inflammatory induction. Specifically, Tey et al. reported that no increase in circulating sEV‐pIgR levels was observed in inflammatory hepatic conditions, namely, chronic HBV infection or HBV‐related cirrhosis [9]. It remains unclear, however, whether sEV‐pIgR arises from an independent biogenesis pathway, altered vesicular trafficking, or a higher molecular threshold for secretion compared with cellular pIgR.

## 3 Prognostic and Diagnostic Potentials of sEV‐pIgR

The study by Tey et al. highlights a paradigm shift in understanding HCC progression and metastasis, suggesting that tumour aggressiveness is not solely attributed to intrinsic genetic changes. Instead, a complex interplay of systemic factors, including sEVs, modulates the tumour microenvironment, influencing tumour aggressiveness and metastatic potential. Clinically, this suggests that highly aggressive HCCs might be identified by their sEV output that prepares metastatic niches. Such biomolecular phenomena could also explain why some patients develop early metastasis despite having small primary tumours [15].

From a diagnostic standpoint, pIgR levels in circulating sEVs were significantly higher in late‐stage HCC patients and tended to decrease after tumour resection, indicating that sEV‐pIgR reflects tumour burden (Table 1). Currently, HCC surveillance relies on imaging and alpha‐fetoprotein (AFP) levels [16], which often miss early or residual disease. sEV analysis could augment detection, as tumour‐derived sEVs are present even at early stages and throughout disease progression. Notably, sEV‐pIgR distinguished HCC patients from those with common benign liver pathologies, such as chronic hepatitis infection and hepatitis related cirrhosis, underscoring its potential specificity for malignancy [9]. If further validated, an elevated sEV‐pIgR could alert clinicians to aggressive tumours or residual disease post‐treatment, enabling earlier intervention. In another study, sEV‐pIgR derived from serum and urine detected in HCC patients effectively distinguished cancer patients from healthy individuals [17]. Combined quantification and cargo analysis of sEVs may serve as a valuable tool for early detection and ongoing monitoring of HCC.

**TABLE 1:** Diagnostic potential and research maturity of pIgR in different types of liver cancers.

Type	Medium	Expression pattern	Diagnostic potential	Maturity	Reference
HCC	Plasma/serum	Elevated sEV‐pIgR, especially late‐stage; declines after curative resections	✓ Discriminates HCC from benign liver disease; ✓ Augment surveillance alongside imaging/AFP; ✓ Aggressiveness signal and post‐op monitoring; ✗ Early‐stage sensitivity remains uncertain	Moderate to high	9
ICC	Plasma/serum	Plasma pIgR elevated; tumour‐cell pIgR demonstrated by immunostaining	✓ Screening candidate in high‐risk settings; ✓ Adjunct diagnostic in symptomatic disease; ✗ Further validation models still required	Moderate	19, 20
cHCC‐CCA	—	No dedicated studies of pIgR expression or sEV‐pIgR identified; biological heterogeneity expected	✗ Research Gap	Low	—

*Note:* ✓ indicates supported by primary evidence/clinically suggestive utility and ✗ indicates unconfirmed evidence or research gap.

## 4 Therapeutic Applications: Targeting sEV‐pIgR

Beyond diagnostics, the study's therapeutic implications are also significant. The profound suppression of tumour growth in mice by combining an anti‐pIgR antibody with sorafenib suggests a treatment strategy to disrupt sEV‐mediated communication [9]. Phenotypically, other studies have also established that a knockdown of pIgR is associated with decreased EMT [18]. It is clear, therefore, that future therapies could involve the blockade of sEV‐mediated signalling, either by neutralising specific sEV components or inhibiting their release and uptake, thereby slowing disease progression. This approach represents a paradigm shift from solely targeting tumour cells alone to also modulating extracellular messengers that coordinate metastasis. Overall, these findings challenge conventional approaches to traditional HCC management and open avenues for innovative sEV‐based diagnostic and therapeutic strategies. Further investigations are thus essential to elucidate the pharmacokinetics and optimal delivery mechanisms for these agents.

## 5 Beyond HCC—Role of sEV‐pIgR in Other Primary Liver Cancers

While HCC is the predominant form of primary liver cancer, it is important to consider the role of sEV‐pIgR in other primary liver malignancies, namely intrahepatic cholangiocarcinoma (ICC) and combined hepatocellular‐cholangiocarcinoma (cHCC‐CCA) (Table 1).

### 5.1 Intrahepatic Cholangiocarcinoma (ICC)

Although ICC originates from biliary epithelial cells (cholangiocytes), which typically have a high baseline of pIgR, emerging evidence suggests that increased levels of pIgR may also be implicated in CCA biology. In the context of malignancy and chronic inflammation, CCA cells can aberrantly express pIgR. A proteomic study by Prasopdee et al. identified pIgR as a potential biomarker for ICC in the context of *Opisthorchis viverrini* (Liver fluke) infection, a known carcinogen [19]. The authors found plasma pIgR levels were significantly elevated in CCA patients compared to both healthy individuals and *O. viverrini* ‐infected individuals, with an ELISA‐based assay achieving approximately 78% sensitivity and 71% specificity for detection. This indicates that tumour‐derived pIgR is released into circulation, making it a candidate screening marker in endemic regions. However, the methodology of this study falls short of specifying whether the plasma pIgR was associated with sEV or not; however, the fact that the study did not use sEV‐depleted serum suggests that it is not without possibility that there was sEV‐pIgR in the serum samples. The deduction that the serum pIgR may be sEV associated can be partially supported by a comprehensive analysis of sEV associated proteins in CCA by Lapitz and colleague, who highlighted sEV‐pIgR among serum markers predictive of ICC development in high‐risk patients. Specifically, elevated sEV‐pIgR in patients with primary sclerosing cholangitis was found to foretell the eventual emergence of CCA [20]. Furthermore, in clinically established ICC cases, immunofluorescence studies have shown pIgR protein within the tumours, co‐localising with CK19‐positive malignant cholangiocyte [20, 21]. Such finding confirms that cholangiocarcinoma cells can express pIgR in response to the inflammatory tumour microenvironment. However, functionally, it remains to be determined whether pIgR actively contributes to cholangiocarcinoma progression as it does in HCC.

### 5.2 Combined Hepatocellular‐Cholangiocarcinoma (cHCC‐CCA)

cHCC‐CCA is a rare primary liver tumour characterised by the coexistence of hepatocytic and cholangiocytic elements within the same lesion. Given the dual composition, the involvement of pIgR in cHCC‐CCA could be complex. To date, there are no published studies specifically evaluating pIgR expression or function in cHCC‐CCA. Nonetheless, by extrapolation, several possibilities can be considered. The hepatocytic component of cHCC‐CCA would be expected to behave like conventional HCC, overexpressing pIgR and secreting pIgR‐rich sEVs, mirroring typical HCC biology. In contrast, the cholangiocarcinoma component might inherently have low baseline pIgR unless tumour‐associated inflammation induces its expression. The net pIgR level in a combined tumour could therefore depend on the proportion of HCC versus CCA differentiation. However, without direct data, this remains speculative, but it highlights a potential heterogeneity, and more research should be dedicated to exploring such possibility.

## 6 Moving Forward: Using sEV‐pIgR as a Primary Liver Cancer Biomarker

The identification of reliable biomarkers remains paramount to improving early diagnosis and subsequent management of primary liver cancers. sEV‐pIgR derived from HCC cells emerges as a promising candidate for addressing this need. Multiple studies now highlight the diagnostic and prognostic capabilities of sEV‐pIgR, providing a basis for its potential clinical translation into liquid biopsy assays.

The primary diagnostic advantage of sEV‐pIgR lies in its detectability in bodily fluids, such as serum [22] and urine [23], through sEV cargo analysis. sEVs, by virtue of their stability and presence in circulation at early disease stages, represent an accessible and minimally invasive source for biomarker discovery. As suggested previously in this review, early proteomic profiling studies by Li et al. established sEV‐pIgR as one among a set of glycoproteins significantly enriched in sEVs from HCC patients, clearly differentiating them from healthy controls. Further extending this concept, Tey et al. showed that circulating sEV‐pIgR levels were significantly elevated in patients with late‐stage HCC relative to early‐stage disease and benign liver conditions such as cirrhosis. Remarkably, the levels of sEV‐associated pIgR demonstrated a notable decrease following successful tumour resection, suggesting a direct correlation with tumour burden and metastatic capability.

Current diagnostic modalities for HCC, such as ultrasound imaging combined with AFP measurements, have limitations in sensitivity and specificity, especially in detecting early‐stage tumours or predicting tumour recurrence [24]. sEV‐based assays, including pIgR quantification, could augment or even outperform these traditional approaches. As Tey et al. illustrated, sEV‐pIgR not only discriminated HCC patients from those with benign hepatic disorders but also provided information regarding the aggressiveness and metastatic potential of the cancer. This characteristic could be highly valuable in clinical settings, enabling clinicians to stratify patient risks more precisely and to initiate appropriate therapeutic interventions more promptly.

Moreover, the specificity of sEV‐pIgR in differentiating primary malignant hepatic conditions from benign liver pathologies enhances its appeal as a biomarker. Tey et al. reported no elevation of circulating sEV‐pIgR in patients with chronic HBV or HBV‐related cirrhosis, in contrast to its significant elevation in HCC [9]. Lapitz et al. further demonstrated that sEV‐pIgR elevation indicated specific differentiation between early CCA and sclerosing cholangitis [20, 25]. For the specific differentiation between the two major types of primary liver cancers, Arbelaiz et al. demonstrated that multi‐protein EV proteomic signatures could distinguish intrahepatic CCA from HCC with an AUC of 0.894 [25]. This indicates that while pIgR alone may not differentiate between these malignancies, its integration into broader panels may enable reliable subtype discrimination.

It is clear that sEV‐pIgR emerges as a highly promising, but preliminary, biomarker candidate for primary liver cancers with significant potential to enhance early detection, monitor tumour aggressiveness, and predict outcomes. By complementing or enhancing existing diagnostic modalities, sEV‐pIgR offers an innovative path towards improved patient management and survival outcomes in primary liver cancer care. pIgR in sEVs might serve as a complementary biomarker when combined with existing clinical markers, forming comprehensive diagnostic panels that improve accuracy and reduce false negatives or positives. For instance, combining sEV‐pIgR measurements with established biomarkers like AFP or novel molecular markers such as circulating tumour DNA (ctDNA) [26] could enhance early detection and more reliably monitor post‐treatment disease progression or recurrence. Such optimism is supported by a study done in 2017 by Haertel et al. which has demonstrated that using large EV as a liquid biopsy marker resulted in an AUC value of 0.7 [27], showing fair discrimination of HCC. With increasing recent evidence and specificity in particular sEV associative protein marker, it is hopeful that the AUC value would eventually reach clinically acceptable levels and, possibly, exceed the current AUC value of AFP in HCC determination—which is generally slightly under 0.8, indicating fair to moderate discrimination [28]. While still lagging in HCC, the diagnostic capability of sEV‐pIgR alone has been demonstrated in CCA—the second most common type of primary liver cancer. Arbelaiz et al. reported that sEV‐pIgR has achieved an AUC of 0.905 with 75% sensitivity and 95% specificity for detecting early‐stage CCA [25]. Despite this, it is still imperative that future studies further investigate the exact AUC values for using sEV‐pIgR alone, and sEV‐pIgR combined with AFP as diagnostic biomarkers for HCC and compare with that of solely using AFP.

## 7 Limitations

While the studies regarding the role of sEV‐pIgR in diagnosing and monitoring cancer are compelling, several limitations and open questions remain before sEV‐pIgR findings can be fully translated to the clinic.

### 7.1 Isolation Complexity and Lack of Standardisation

Isolating sEVs typically requires ultracentrifugation, a labour‐intensive process that demands specialised equipment and expertise, making it impractical for routine clinical diagnostics. Simpler methods, such as polymer‐based precipitation, can increase sEV yield but often sacrifice purity, co‐isolating non‐sEV proteins and RNA contaminants [29]. It is obvious that it is impossible to obtain and purify high quality sEV in a clinical setting. To date, there is no universally accepted protocol for sEV collection and processing, resulting in significant variability between studies. Without streamlined, standardised methods, the integration of sEV assays into clinical practice remains a challenge.

### 7.2 Early Detection Capability

The data strongly link sEV‐pIgR to late‐stage HCC, but its utility in early‐stage disease is less certain. In the study, early HCC patient sEVs had only modest pIgR increases (often not statistically significant vs. normal). It is doubtful if sEV‐pIgR could be utilised to detect small, early‐stage tumours requiring prompt intervention as it only shows significant elevation as tumours grow larger and become invasive. Improving assay sensitivity or combining sEV‐pIgR with other sEV markers (e.g., sEV‐DNA mutations or other proteins) might be required for robust early detection. Furthermore, to date, no study has directly compared the diagnostic performance of sEV‐pIgR against AFP combined with imaging for early‐stage HCC detection, and this remains a critical gap requiring prospective validation.

### 7.3 Gaps in Clinical Translation

Although research on using pIgR as biomarkers is advancing rapidly, there remain significant gaps in clinical validation. Most published studies are exploratory or limited to small patient cohorts. Large‐scale, prospective clinical trials demonstrating the diagnostic or prognostic accuracy of sEV‐based tests for HCC are lacking. To date, there exists only one blood test that uses EV as a biomarker for detecting early stage HCC that is granted an FDA Breakthrough Device Designation and is undergoing further large scale validation studies [30]. As a result, there currently exists no widely adopted regulatory approved sEV‐based assay for routine use in early HCC detection. Until standardised assays are widely available, sEV cargos are likely to remain research tools rather than routine clinical diagnostics.

### 7.4 Therapeutic Target Challenges

Using an antibody to block sEV‐pIgR function showed efficacy in mice, but safety and delivery in humans require caution. pIgR is expressed in normal tissues (e.g., intestinal mucosa), so systemic anti‐pIgR therapy could, in theory, interfere with mucosal immunity (affecting IgA transport and gut defence). Targeted delivery of such antibodies to the tumour or careful dosing will require optimisation through clinical trials. Moreover, completely halting sEV release or uptake in patients is currently impractical. It's unclear if partial inhibition of single sEV cargo will be effective in slowing HCC progression in human patients. Thus, while conceptually sound, an sEV‐pIgR–targeted therapy needs much more evaluation (pharmacokinetics, toxicity, and efficacy in more heterogeneous tumour models).

## 8 Conclusion and Perspectives

The identification of pIgR as a functionally active component of sEVs in primary liver cancer represents a major advancement in our understanding of tumour biology and opens new avenues for diagnosis and treatment. Rather than serving merely as a mucosal immune receptor, sEV‐pIgR is now recognised as an oncogenic driver that promotes cancer stemness, metastasis, and therapeutic resistance through sEV‐mediated signalling pathways PDK1/Akt/β‐catenin. This sEV‐associated pIgR, especially when elevated in patient serum, is a direct reflection of tumour aggressiveness and metastatic potential.

From a clinical standpoint, sEV‐pIgR offers promise as a non‐invasive biomarker for primary liver cancer surveillance. Its detectability in circulation, correlation with disease stage, and decline post‐surgical resection underscore its potential utility in diagnosis, monitoring, and recurrence prediction. Compared to traditional markers such as AFP, sEV‐pIgR may show better specificity for primary liver cancers and outperform current standards when used alone or as part of a multi‐analyte panel. Furthermore, its presence in ICC and possible relevance in cHCC‐CCA point to broader diagnostic applicability across primary liver cancers.

Therapeutically, targeting sEV‐pIgR using neutralising antibodies or inhibition of sEV release may augment existing HCC treatments like sorafenib, particularly in aggressive or drug‐resistant cases. However, challenges remain: technical barriers in sEV isolation, lack of standardised protocols, and limited data on early‐stage sensitivity hinder immediate clinical translation. Large‐scale validation and assay development are necessary steps forward.

In summary, sEV‐pIgR bridges fundamental tumour biology with clinical relevance. As both a biomarker and therapeutic target, it exemplifies the growing promise of sEV‐based oncology. With further refinement and validation, sEV‐pIgR may soon become a powerful tool in the early detection, risk stratification, and treatment of primary liver cancer, transforming how clinicians understand and manage this deadly disease.

## Author Contributions

Conceptualisation was performed by J.Y.W.P.; Writing – original draft by J.T.R.H.; Writing – review and editing was carried out by J.T.R.H., S.W.K.W. and J.Y.W.P.; Supervision and funding acquisition were provided by J.Y.W.P.

## Funding

This work was funded by the National Natural Science Fund under National Natural Science Foundation of China (Project No. 82573164), HKUMed Research Collaboration Booster Fund, The University of Hong Kong.

## Conflicts of Interest

The authors declare no conflicts of interest.

## Data Availability

Data sharing not applicable to this article as no datasets were generated or analysed during the current study.
